# Role of Feature Diversity in the Performance of Hybrid Models—An Investigation of Brain Tumor Classification from Brain MRI Scans

**DOI:** 10.3390/diagnostics15151863

**Published:** 2025-07-24

**Authors:** Subhash Chand Gupta, Shripal Vijayvargiya, Vandana Bhattacharjee

**Affiliations:** Department of CSE, Birla Institute of Technology, Ranchi 835215, India; sgupta@bitmesra.ac.in (S.C.G.); svijayvargiya@bitmesra.ac.in (S.V.)

**Keywords:** brain tumor, deep learning, VGG16 model, DensetNet121 model, ResNet50 model, feature fusion

## Abstract

**Introduction**: Brain tumor, marked by abnormal and rapid cell growth, poses severe health risks and requires accurate diagnosis for effective treatment. Classifying brain tumors using deep learning techniques applied to Magnetic Resonance Imaging (MRI) images has attracted the attention of many researchers, and specifically, reducing the bias of models and enhancing robustness is still a very pertinent active topic of attention. **Methods**: For capturing diverse information from different feature sets, we propose a *Features Concatenation-based Brain Tumor Classification (FCBTC) Framework using Hybrid Deep Learning Models.* For this, we have chosen three pretrained models—ResNet50; VGG16; and DensetNet121—as the baseline models. Our proposed hybrid models are built by the fusion of feature vectors. **Results**: The testing phase results show that, for the FCBTC Model-3, values for Precision, Recall, F1-score, and Accuracy are 98.33%, 98.26%, 98.27%, and 98.40%, respectively. This reinforces our idea that feature diversity does improve the classifier’s performance. **Conclusions:** Comparative performance evaluation of our work shows that, the proposed hybrid FCBTC Models have performed better than other proposed baseline models.

## 1. Introduction

Brain tumors are abnormal growths of tissue in which cells multiply without control and indefinitely. They are among the most aggressive and life-threatening conditions affecting both children and adults, often requiring prompt and accurate diagnosis to improve treatment effectiveness. Magnetic Resonance Imaging (MRI) is one of the most widely used imaging techniques in brain tumor diagnosis due to its non-invasive nature and ability to produce detailed images of brain structures. However, interpreting MRI scans manually can be time-consuming, subjective, and prone to errors, especially given the complexity and variability of brain tumors. To overcome these limitations, deep learning has emerged as a promising solution for the automated detection and classification of brain tumors visible in brain MRI images. Convolutional Neural Networks (CNN) deep learning models are particularly effective, as they can learn intricate patterns directly from MRI images without relying on handcrafted features. These models are capable of accurately distinguishing between different types of brain tumors, including gliomas, meningiomas, and pituitary tumors.

**Literature Review:** From the several research papers, it has been observed a significant advancement in recent years in the field of brain tumor detection and classification using deep learning. Several computer science researchers have proposed diverse Models based on Convolutional Neural Network (CNN), Vision transformer, and hybrid architectures based on approaches to enhance the diagnostic accuracy and computational efficiency.

Dhakshnamurthy et al. [1] used transfer learning models to enhance classification performance in limited datasets, while Mahmud et al. [2] performed a comparative study using various deep networks for MRI-based tumor detection. Nayak et al. [3] demonstrated the efficacy of Dense EfficientNet in improving classification accuracy. Similarly, De Benedictis et al. [4] proposed a novel combination of topological data analysis and tensor decomposition to enhance MRI-based tumor classification. Deep segmentation networks have also been explored extensively. Mostafa et al. [5] implemented deep learning-based segmentation models on MRI images, while Abdusalomov et al. [6] utilized several CNN variations for tumor localization. ZainEldin et al. [7] integrated deep learning with Sine-Cosine Grey Wolf Optimization for feature selection and classification. Futrega et al. [8] presented a U-Net variant optimized for glioma segmentation.

In classification-focused studies, Younis et al. [9] used ensemble learning with VGG-16 for performance improvement, and Zahoor et al. [10] proposed a hybrid deep and ensemble learning model. Sultan et al. [11] built a fully optimized CNN framework for multiclass tumor detection, and Al-Galal et al. [12] provided a systematic review of deep learning in MRI tumor classification. Rahman et al. [13] introduced an IoT-based real-time brain tumor detection system.

Patil et al. [14] discussed recent updates on segmentation using deep learning, while Jiang et al. [15] introduced SwinBTS, a 3D transformer-based model for multimodal MRI segmentation. Yeo et al. [16] reviewed deep learning algorithms for intracranial hemorrhage detection, highlighting transferable techniques for tumor detection. Earlier methods by More et al. [17,18] utilized CNNs and DNNs for tumor classification. Wozniak et al. [19] explored correlation learning in CT-based detection. Raj et al. [20] proposed BrainNET, a deep learning network for MRI tumor classification. Anilkumar and Kumar [21] combined block-wise fine-tuning with KNN for improved performance.

Boustani et al. [22] and Deepak and Ameer [23] both used CNNs and transfer learning strategies for MRI classification. Maqsood et al. [24] integrated multimodal data using a deep network and SVM classifier. Kokkalla et al. [25] used Dense Inception Residual networks for three-class classification, while Diaz-Pernas et al. [26] applied multiscale CNNs for classification and segmentation. Sharif et al. [27] proposed a framework based on YOLOv2 and CNN for tumor localization, and Rizwan et al. [28] implemented a Gaussian CNN for glioma grading. Rasool et al. [29] introduced a fine-tuned SqueezeNet with SVM for classification tasks. Gumaei et al. [30] used a hybrid feature extraction method with a regularized ELM classifier for efficient tumor detection.

Badza and Barjaktaric [31] employed standard CNN architecture for MRI-based classification. Polat and Gungen [32] applied deep transfer learning for efficient tumor recognition. Çinar and Yildirim [33] introduced a hybrid CNN architecture for improved detection accuracy, and Obeidavi and Maghooli [34] focused on residual CNNs for robust MRI tumor classification. Further interdisciplinary comparisons were drawn by Kumaraswamy et al. [35], who applied CNN ensembles to breast cancer classification, indicating architectural transferability. Karamehic & Jukic [36] presented a VGG-16–based approach enhanced by using the Python 3.11.7 Imaging Library (PIL) for preprocessing. From an optimization standpoint, Kingma & Ba’s Adam optimizer [37] remains a cornerstone choice for training deep neural networks efficiently. Foundational deep learning methodologies underpinning such models include Simonyan & Zisserman’s VGG16 architecture [38] and He et al. [39] with their ResNet architecture, which introduced residual connections to mitigate vanishing gradients and enabled training of significantly deeper networks—pivotal for medical imaging applications requiring deep representations. Huang et al. [40] proposed the DenseNet model, introducing densely connected convolutional layers to improve information flow and reduce parameter redundancy. Kumar et al. [41] conducted an empirical study comparing handcrafted and dense feature techniques for cancer classification, offering insights into feature extraction strategies.

**Motivation and Contribution:**

We find that most papers in the reference list have not applied the features concatenation technique except reference [10]. So, we have identified research gaps as taking features from the different CNN base models and concatenating them to build the hybrid models and study the role of feature diversity in the performance of the hybrid models.

So, with an aim to improve the diagnosis of diseases, in particular, brain tumor classification, this study takes up the following research objectives:

- To create baseline models by fine-tuning pretrained models to adapt them for Brain Tumor classification.
- To concatenate feature sets from these baseline models and build hybrid architectures with modified classification heads.
- To validate all the models on benchmark datasets.

To achieve the above objectives, we have chosen three pretrained models—ResNet50 [38]; VGG16 [39]; and DenseNet121 [40]—as the baseline models. The dataset used in this study is publicly available on Kaggle [42].

For this, we propose a *Features Concatenation-based Brain Tumor Classification (FCBTC) Framework using Hybrid Deep Learning Models.* The key idea is to enhance the model’s capability to capture complex patterns by feature concatenation from data sets. Secondly, we also want to enhance the representational power by combining feature sets.

**Key contributions of the paper are:**

- Fine-tuning of ResNet50, VGG16, and DenseNet121 baseline models to adapt them for brain tumor classification.
- Concatenating different feature sets, enabling the model to learn more expressive representations, leading to better generalization and improved performance on various tasks.
- Building a hybrid model by feature concatenation and a modified classification head, with the baseline models fine-tuned on the training set and evaluating its performance.
- Performance evaluation on test dataset to show the efficacy of our proposed models and comparison analysis.

## 2. Materials and Methods

### 2.1. Dataset Description and Pre-Processing

**Figure 1:**
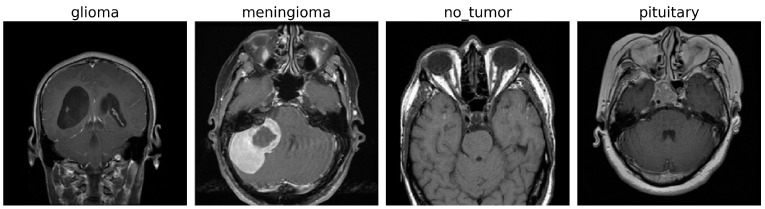
MRI Images of Brain Tumor and Normal Brain.

This section discusses the brain tumor dataset [42] that has been used in this work and its preprocessing. The data set used in this work consists of MRI brain scans. A sample of the four categories of images shown in Figure 1.

The dataset is contained in the two main directories: Training and Testing. Each of these directories contains four subfolders corresponding to the respective tumor classes: No Tumor, Glioma, Meningioma, and Pituitary Tumor. The image counts of all classes are given in Table 1. This organized structure facilitates supervised learning by providing clearly labeled data for both the training and the evaluation phases of the deep learning models.

The size of an image is given as W × H × C. Here W denotes width, H denotes height, and C denotes the number of color channels. E.g., the size notation 224 × 224 × 3 refers to the width and height of 224 pixels, and the number 3 indicates the 3 color channels corresponding to Red, Green, and Blue. In fact, W × H indicates the spatial resolution of an image. In the dataset used here, the original width-wise and height-wise sizes of most images vary between 200 × 200 and 512 × 512, and all images have the same 3 color channels.

In the dataset the training and testing data are already separated, so we have used the dataset in this work as follows:

For the training phase of all the models, we have applied a common split over the training data as 80% for training and 20% for validation during training and validation check.

For the testing phase of all the models, we have used complete testing data, which is separately available in the testing data folder.

### 2.2. Pre-Processing

To ensure the dimensional compatibility of images, adjustments for data imbalance and model performance, and several pre-processing steps have been performed. These steps include image resizing, normalization of image pixel values, and adjusting imbalance of data.

#### 2.2.1. Image Resizing

In the dataset used here, the original width-wise and height-wise size of most images varies between 200 × 200 and 512 × 512, and all images have the same 3 color channels. The baseline DL models and hybrid DL models used in this paper are based on pretrained VGG16, ResNet50, and DenseNet121. These pretrained models are trained on the ImageNet dataset, which consists of images standardized to a size of 224 × 224 × 3. Feeding the models with images of a different size can lead to shape mismatch errors or suboptimal performance. To ensure compatibility with these architectures, we have applied the image resizing transformation on our dataset instead of image cropping. In image resizing, we have used Bi-linear interpolation, which retains the complete image but at a different resolution. While in the case of image cropping, it results in loss of information.

#### 2.2.2. Image Normalization

Normalization is a key pre-processing step that adjusts the pixel values of input images to a consistent scale. This standardization improves both the training stability and convergence of deep learning models. The benefits of normalization include

- Preventing issues like exploding or vanishing gradients during backpropagation.
- Enhancing the efficiency and stability of optimization algorithms such as SGD and Adam, etc.
- Ensuring consistent learning across mini-batches.
- Reducing bias towards features with higher magnitudes, improving generalization.

In this paper, image normalization is performed using the standard mean and standard deviation values given below:

- Mean: [0.485, 0.456, 0.406]
- Standard Deviation: [0.229, 0.224, 0.225]

These values are based on the ImageNet dataset statistics and have been used in pretrained models like ResNet50, Vgg16, DenseNet121, etc.

#### 2.2.3. Adjustment of Data Imbalance

In many real-world datasets, especially in medical imaging, class imbalance is a common issue where certain categories have significantly more samples than others. This imbalance can bias the model toward the majority class, resulting in deceptively high overall accuracy but poor performance in detecting minority classes, which are often the most clinically important. To address this issue, we have applied a resampling technique using WeightedRandomSampler, a utility provided by the PyTorch framework.

### 2.3. Description of Baseline Deep Learning Models

This section outlines the architectural design of the deep learning models used for image classification, along with the setup for training, validation, and testing. To analyze and classify the brain tumor MRI images, we have applied three fine-tuned pretrained CNN models named VGG16, ResNet50, and DenseNet121. These were selected as base models based on their architectural design diversity, good feature extraction capabilities, and performance on medical imaging tasks. These are widely used in medical image analysis. The rationales for choosing these models are as follows:

**VGG16**: It has a simple and uniform design with only 16 layers and employs a consistent pattern of 3 × 3 convolutional filters that make it capable of extracting detailed spatial features. 

**ResNet50**: This model architecture includes residual connections that address the vanishing gradient problem, which makes it capable of training deeper networks effectively and suitable for identifying tumor structures.

**DenseNet121**: The dense connectivity of the DenseNet121 model ensures efficient feature propagation as each layer receives inputs from all previous layers, improving feature reuse, which helps in preserving information across layers and enhances gradient flow.

Since we have used three fine-tuned pretrained models, which were previously trained on the large-scale ImageNet dataset. By using these pretrained models, we have transferred their pre-learning and carried out fine-tuning to train on our datasets, which is known as transfer learning with fine-tuning.

In our experimentation, we have used transfer learning using fine-tuned pretrained base models, which are specifically designed to perform well even with smaller datasets by leveraging knowledge learned from large-scale datasets. This approach allows the model to generalize better despite limited data availability.

Furthermore, the concatenation of different feature sets was intended to enrich the representation learned by the model, which complements the benefits of transfer learning.

#### 2.3.1. Overview of Architectural Design of ResNet50 CNN Model

**Figure 2:**
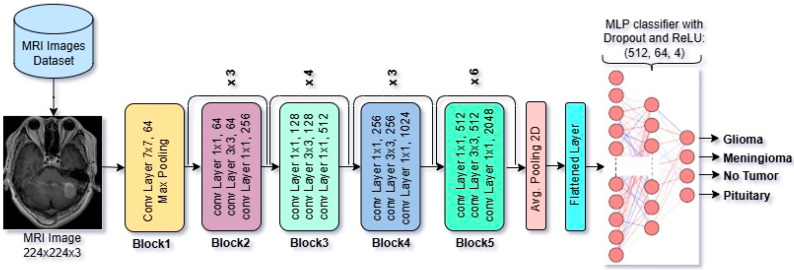
Architectural design of fine-tuned ResNet50 CNN model.

ResNet50 is a widely recognized deep convolutional neural network with 50 layers, originally introduced by Microsoft Research in 2015. Figure 2 shows the architectural diagram of this model. The ResNet50 model is pre-trained on large-scale datasets such as ImageNet. It accepts input images of 224 × 224 × 3. In our case, we have fine-tuned and modified its MLP classifier part to make it capable of performing the classification task on Brain MRI Images. One of the main features is the use of skip (residual) connections, allowing gradients to propagate through the network more effectively during backpropagation.

#### 2.3.2. Overview of Architectural Design of VGG16 Model

**Figure 3:**
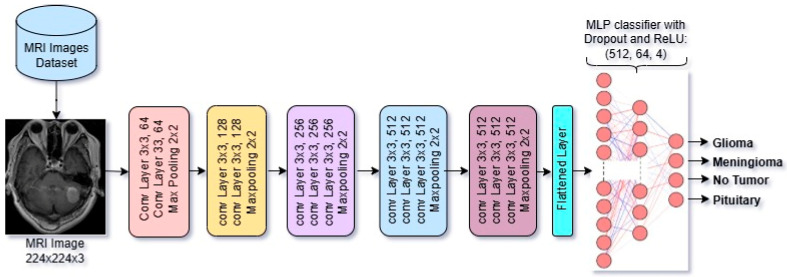
Architectural design of fine-tuned VGG16 CNN Model.

VGG16 is a deep CNN developed by the Visual Geometry Group at the University of Oxford. The pretrained VGG16 model contains 16 weight layers, in which 13 layers are convolutional layers and 3 are fully connected layers, and uses ReLU activation throughout. It accepts input images of 224 × 224 × 3. In our case, we have fine-tuned and modified its MLP classifier part to make it capable of performing the classification task on Brain MRI Images. VGG16 contains approximately 138 million parameters, making it computationally intensive but highly effective for image recognition tasks. VGG16 is widely used for transfer learning and feature extraction in computer vision applications. The model is shown in Figure 3.

#### 2.3.3. Overview of Architectural Design of DenseNet121 Model

**Figure 4:**
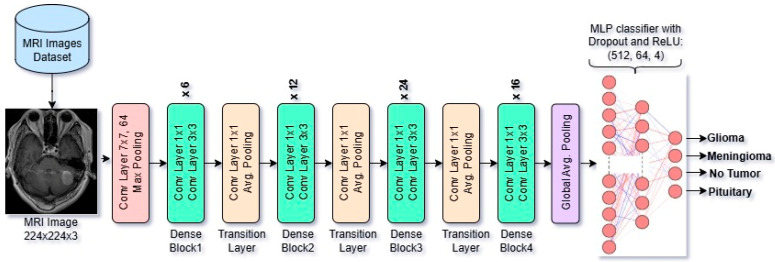
Architectural design of fine-tuned DenseNet121 Model.

DenseNet121 is a deep convolutional neural network of the DenseNet family. Due to its dense connectivity, where each layer receives inputs from all previous layers, it is known as DenseNet. It consists of 121 layers, including 4 dense blocks with 1 × 1 and 3 × 3 convolutions, followed by global average pooling (GAP) and a fully connected layer for classification. The model has approximately 8 million parameters. It is also pretrained on the ImageNet Dataset. It accepts input images of 224 × 224 × 3. In our case, we have fine-tuned and modified its MLP classifier part to make it capable of performing the classification task on Brain MRI Images. DenseNet-121 is computationally efficient due to feature reuse, reducing redundancy and improving gradient flow. It is widely used in image classification and medical imaging applications. The model is shown in Figure 4.

#### 2.3.4. Features Concatenation-Based Brain Tumor Classification (FCBTC) Framework

The proposed Features Concatenation-based Brain Tumor Classification (FCBTC) Framework is presented.

**Figure 5:**
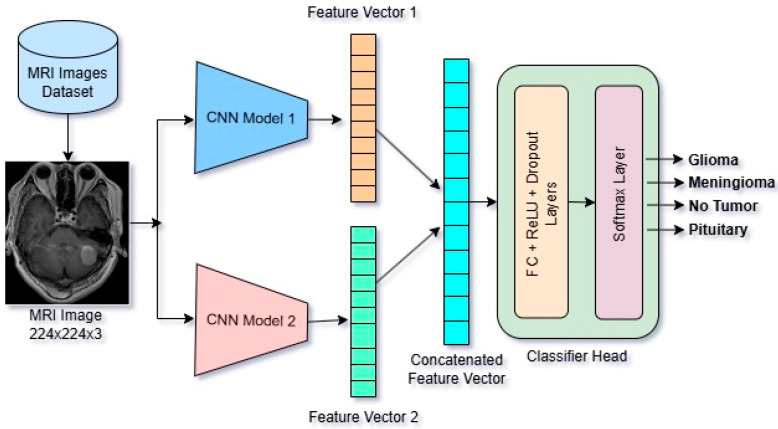
Schematic diagram of Features Concatenation-based Brain Tumor Classification (FCBTC) Framework.

The schematic diagram of the Features Concatenation-based Brain Tumor Classification (FCBTC) Framework is presented graphically in Figure 5 and algorithmically by the Algorithm 1. In our work, besides the three baseline Models, we have proposed three more FCBTC hybrid models named FCBTC Model-1 (VGG16 + ResNet50), FCBTC Model-2 (DenseNet121 + VGG16), and FCBTC Model-3 (ResNet50 + DenseNet121).


**Algorithm 1.** Features Concatenation-Based Brain Tumor Classification (FCBTC) Framework
Fine-tune baseline models M1 and M2 with datasetExtract feature vectors f1 and f2 from M1 and M2 after flattening layer.Perform concatenation of features: f_con = f1 ‖ f2The concatenated feature vector is fed into a MLP head z = MLP(f_con)Final classification by SoftMax layer c = Softmax(z)



In the CNN models, the feature vector dimensions are determined by the architecture of each model’s output of the final convolutional block and pooling layer just before the classification. ResNet50 increases the number of filters progressively, reaching 2048 in the final block. In ResNet50, the final convolutional and pooling output is a feature map of shape 1 × 1 × 2048, which, after flattening, results in a 2048-dimensional feature vector. In VGG16, the final convolutional and pooling output is a feature map of shape 7 × 7 × 512, which, after flattening, results in a 25,088-dimensional feature vector. DenseNet121, due to its parameter-efficient design and feature reuse, ends with 1024 filters. In DenseNet121, the final dense block outputs a feature map of size 7 × 7 × 1024. And after applying Global Average Pooling, this results in a 1024-dimensional feature vector. So, the dimensional difference of feature vectors of VGG16, ResNet50, and DenseNet121 occurs from the design of each model architecture.

In FCBTC Model-1 (VGG16 + ResNet50), the concatenated feature vector size is 27,136 (25,088 + 2048). In FCBTC Model-2 (Densenet121 + VGG16), the concatenated feature vector size is 26112 (1024 + 25,088). In FCBTC Model-3 (Densenet121 + Resnet50), the concatenated feature vector size is 3072 (1024 + 2048). In these hybrid models, their component base models feature vector sizes and architectural parameters that are different.

**Figure 6:**
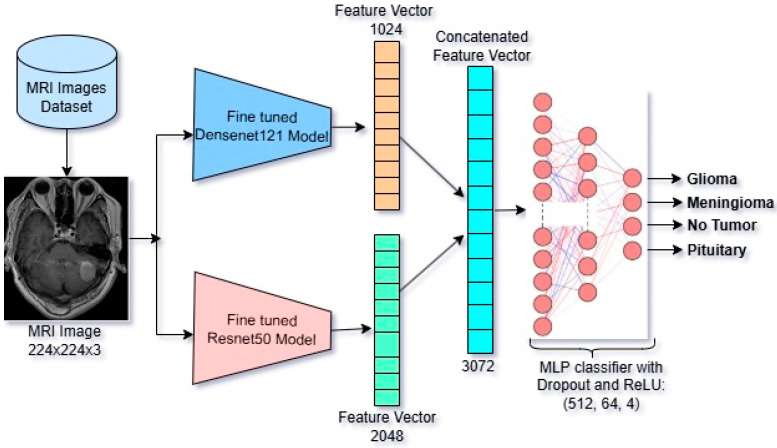
FCBTC Model-3 with ResNet50 + DenseNet121.

A particular materialization of the FCBTC framework with DenseNet121 and ResNet50 trained on the full dataset is shown in Figure 6.

#### 2.3.5. Training, Validation, and Testing Setup of Learning Models

In this paper, for training of all models, we have applied a common split of training data as 80% for training and 20% for validation during training. The testing dataset is separate for testing. To adapt these models to learn domain-specific features from MRI image data and classification tasks, we have applied the common strategy for fine-tuning, modification in the classifier part, and proposed hybrid architectural modifications based on feature concatenation. We have proposed the MLP classifier with a three-linear-layer structure consisting of 512, 64, and 4 neurons or units in each respective layer. The selection of this MLP structure is based on empirical testing and common practices in deep learning to balance model complexity, computational efficiency, and classification accuracy. The rationale for each layer is as follows:

**First Layer (512 units):** This layer serves as the first layer to the MLP, which receives the concatenated feature vector from the hybrid CNN models and maps it to a size of 512. Size 512 was selected to reduce the dimensionality of the high-dimensional concatenated feature vectors output from FCBTC models (e.g., 2048 + 1024 = 3072 in the case of FCBTC Model-3), while preserving enough information for learning. It also acts as a bottleneck layer to prevent overfitting and reduce computational cost.

**Second Layer (64 units):** This is a hidden layer that further transforms the reduced features vector of size 512 to a more compact and abstract representation of smaller size 64, which was chosen to encourage feature compression and introduce non-linearity between the input and output. In fact, the choice of 64 is a trade-off between performance and simplicity—too many units could lead to overfitting; while too few may underfit.

**Output Layer (4 units):** This is the final layer and consists of 4 units, corresponding to the number of brain tumor classes in our classification task.

Besides this, we have taken some wisely chosen hyperparameters and other functions like loss functions and optimization functions.

##### Fine Tuning, Classifier Modification, and Hybrid Architectural Design

- Unfreeze the last six convolutional layers of all the base CNN models—VGG16; ResNet50; and DenseNet121. This allows these layers to be trainable and to better capture high-level features that are critical for distinguishing between different tumor types.
- Replace the classifier part of all the base CNN models by the following multi-layer perceptron (MLP) designed for four output classes. The modified classifier includes the following layers:
  - A fully connected (Linear) layer reducing the dimensionality from input features (features extracted from model’s CNN part) to 512 output neurons.
  - A dropout layer with a dropout rate of 0.5 to mitigate overfitting.
  - A linear layer that maps the 512 features to 64 output neurons.
  - ReLU activation functions in each linear layer to introduce non-linearity.
  - A dropout layer with a dropout rate of 0.5 to mitigate overfitting.
  - A final linear layer that maps the 64 features to 4 output neurons, corresponding to the four brain tumor classes, with a SoftMax function.

##### Selection of Hyperparameters, Loss Function, and Optimization Function

In our experiments, we have used the following hyperparameters and utility functions to train our DL models.

- **Learning rate (α):** The learning rate is the crucial hyperparameter in the matured convergence of the learning model. In our experiment, we have chosen the learning rate 0.0005.
- **Number of epochs**: 30
- **Batch Size (N)**: 64
- **Loss function:** In our classification task, there are four classes in which MRI data are categorized, so there is a need to use a loss function that could penalize the wrong predictions with higher loss values, especially when the predicted probability for the correct class is low. For this reason, we have used crossEntropyLoss() function that is defined as
  (1) $$L\left(\theta \right)=-\frac{1}{N}\sum_{n=1}^{N}\sum_{i=}^{C}y_{n,i}\log\left(p_{n,i}\right)$$
  where,
  $y_{n,i}$ is the true label
  $p_{n,i}$ is the predicted probability for class i.
  *N* is the batch size (in our case, it is 64)
  C is the numbers of classes (in our case, it is 4)
- **Optimization function:** To minimize the loss function, the proposed DL models have used the Adam [37] optimization function with the error backpropagation algorithm in the multilayer perceptron classifier head. The function to update the weights is defined as
  (2) $$\theta_{t+1}=\theta_{t}+\Delta \theta_{t}$$
  (3) $$m_{t}=\beta_{1}*m_{t-1}-\left(1-\beta_{1}\right)*g_{t}\;$$
  (4) $$s_{t}=\beta_{2}*s_{t-1}-\left(1-\beta_{2}\right)*g_{t}^{2}$$
  (5) $$\Delta \theta_{t}=-\alpha \frac{m_{t}}{\sqrt{s_{t}+\varepsilon }}*g_{t}$$
  where,
  $\theta_{t}\;$ are Model parameters at time t.
  α is Learning rate
  $\beta_{1},\;\beta_{2}$ are hyperparameters to control the **exponential decay rates** for the moving averages of the gradients, and the default values taken as 0.9 and 0.999.
  ε is A small positive constant (e.g., 10^−8^) used to avoid division by zero
  $g_{t}$ is gradient with respect to θ_t_
  $m_{t}$ is first moment
  $s_{t}$ is second moment

#### 2.3.6. Hardware Environment Setup

To train CNN-based deep learning models for complex computer vision tasks like image classification requires substantial computational power and memory. Standard CPU-based machines are typically inadequate for such resource-intensive operations. To meet these requirements, we have utilized the Google Colab Pro cloud platform, which offers access to high-performance hardware accelerators. Colab Pro provides a variety of options, including A100 GPUs, L4 GPUs, T4 GPUs, V5e-1 TPUs, and V2-8 TPUs. We have specifically employed the NVIDIA A100 GPU, which offers powerful capabilities suitable for large-scale deep learning tasks. The system configuration used for our training sessions included system RAM: 83 GB and GPU RAM: 40 GB. This environment ensured efficient training, reduced runtime, and enabled the handling of MRI image data without memory bottlenecks.

## 3. Results and Analysis

This section presents the experimental evaluation of the six fine-tuned deep learning models. The first three baseline models are VGG16, ResNet50, and DenseNet121, and the other three hybrid models are FCBTC Model-1, FCBTC Model-2, and FCBTC Model-3. All models have used the same Brain MRI image dataset for the tumor classification task. The assessment includes quantitative performance metrics, training and validation curves, as well as detailed classification reports and confusion matrices for testing phases.

### 3.1. Description of Performance Metrics

To evaluate the effectiveness of the proposed deep learning models for brain tumor classification, we have used widely accepted performance metrics: Accuracy, Precision, Recall, and the F1 Score. These metrics provide a comprehensive understanding of model behavior, particularly in the context of imbalanced medical datasets where false negatives can have serious consequences. The evaluation metrics are computed based on the following classification outcomes for each tumor class:

- **TP (True Positive):** The model correctly predicts a sample as belonging to the class.
- **TN (True Negative):** The model correctly predicts a sample as not belonging to the class.
- **FP (False Positive):** The model incorrectly predicts a sample as belonging to the class.
- **FN (False Negative):** The model fails to predict a sample that actually belongs to the class.

The performance metrics are defined as follows:

***Accuracy:*** Number of correct predictions divided by the total number of samples. It can be calculated as

(6) $$\;Accuracy=\frac{TN+TP}{TP+TN+FP+FN}$$

***Precision:*** How many predicted positives are actually correct. It is calculated as

(7) $$\;\;Precision=\frac{TP}{TP+FP}$$

***Recall:*** How many actual positives were correctly predicted. It can be calculated as

(8) $$Recall=\frac{TP}{TP+FN}$$

***F1-Score:*** Weighted harmonic mean of Precision and Recall. It is calculated as

(9) $$F1\_Score=\frac{2*Precision*Recall}{Precision+Recall}$$

### 3.2. Epoch-Wise Training and Validation Curves

**Figure 7:**
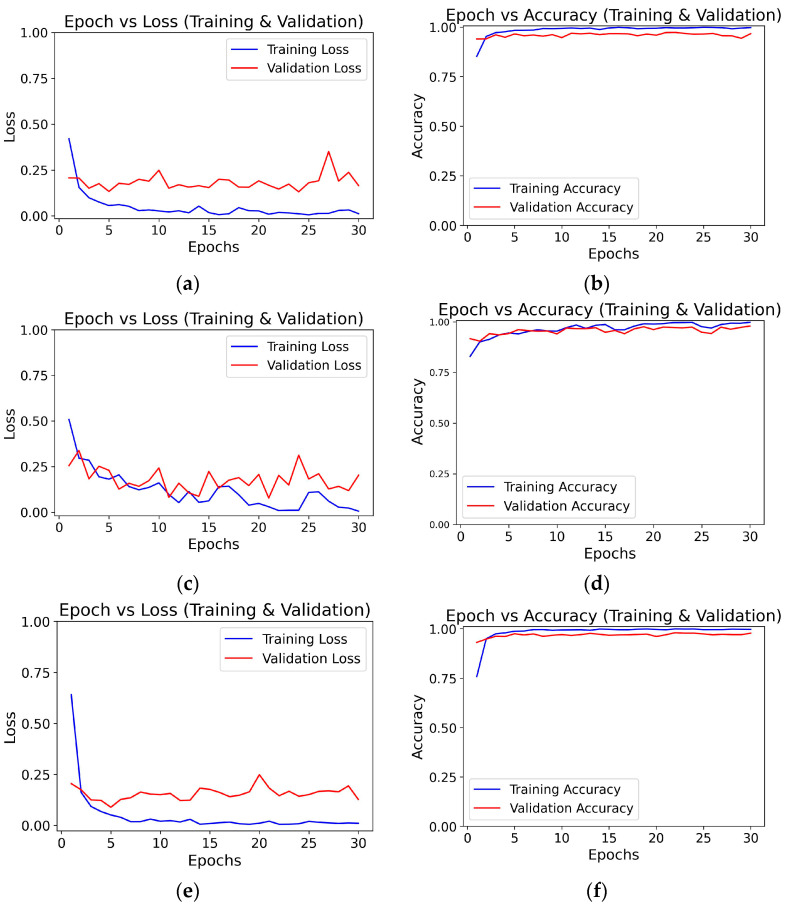
Graph for Epoch vs. Loss and Epoch vs. Accuracy: (**a**,**b**) for ResNet50 model, (**c**,**d**) for VGG16 Model, and (**e**,**f**) for DenseNet121 Model.

**Figure 8:**
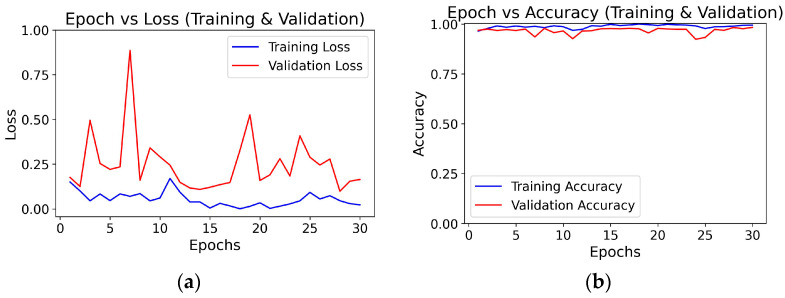

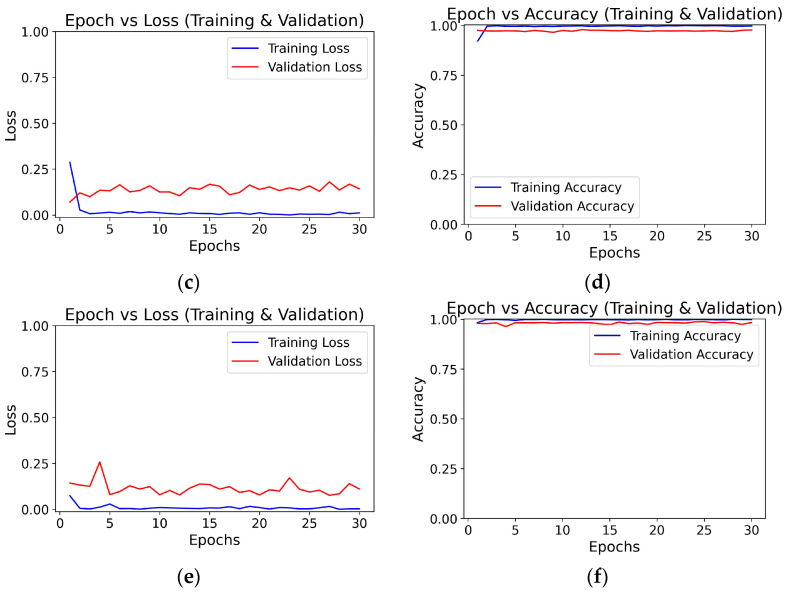
Graph for Epoch vs. Loss and Epoch vs. Accuracy: (**a**,**b**) for FCBTC Model-1, (**c**,**d**) for FCBTC Model-2, and (**e**,**f**) for FCBTC Model-3.

To monitor the learning behavior and convergence of both deep learning models during training, we generated plots of epoch-wise training and validation loss as well as training and validation accuracy. These visualizations are critical for understanding how well the models generalize to unseen data and whether they are overfitting or underfitting. Figure 7 and Figure 8 present the Epoch-wise graphs for the three baseline models and three FCBTC hybrid models, respectively. The training curves illustrate how the models’ prediction error (loss) decreases and accuracy improves over time with each epoch. A consistent decline in validation loss alongside rising accuracy indicates stable learning, while any widening gap between training and validation curves indicates the potential for overfitting.

Validation loss is calculated over fewer samples and is not used to update the model. Small fluctuations or noise in validation batches can lead to loss spikes that smooth out over time.

Similar to Figure 7, the validation loss is calculated over fewer samples and is not used to update the model. Small fluctuations or noise in validation batches can lead to loss spikes that smooth out over time.

### 3.3. Classification Performance Report and Comparison

Following the completion of 30 training epochs for each deep learning model, we have evaluated their performance on the testing datasets. This evaluation involved generating detailed classification reports—including precision; recall; F1-score; and overall accuracy—as well as confusion matrices that provide a class-wise breakdown of correct and incorrect predictions.

#### 3.3.1. Confusion Matrices for Testing Phase of Baseline Models and FCBTC Hybrid Models

**Figure 9:**
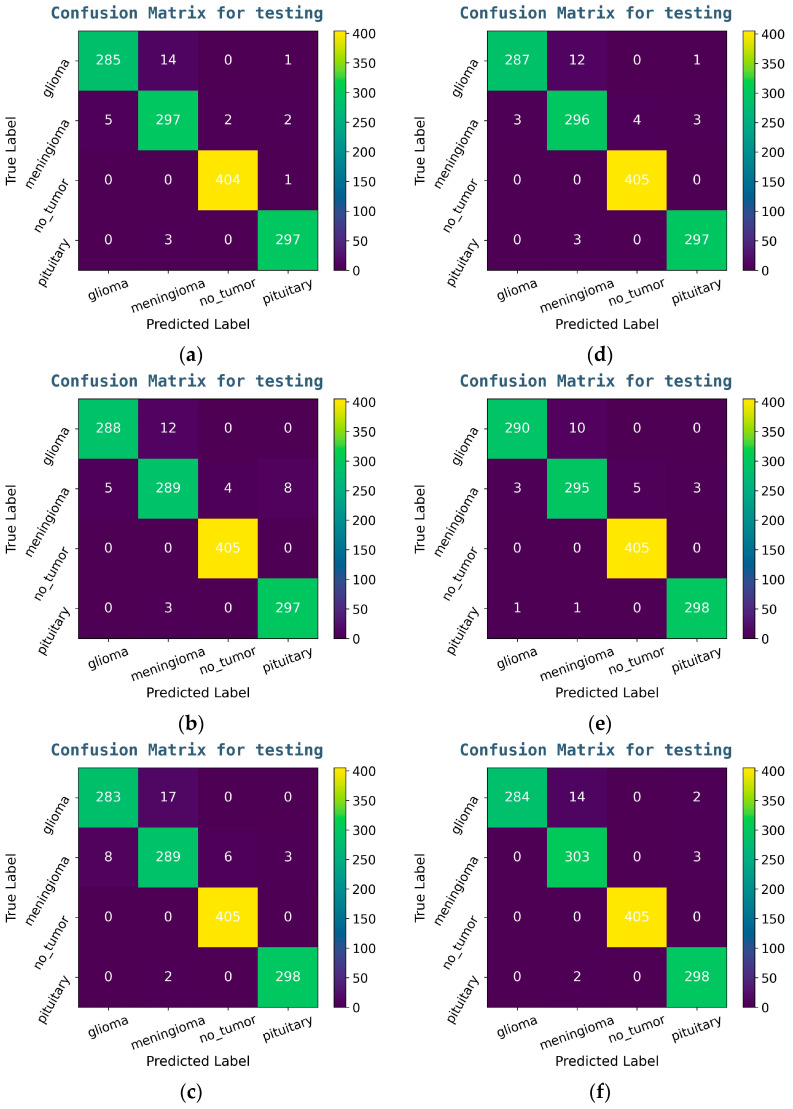
Confusion Matrix for testing Phase: (**a**) for ResNet50, (**b**) for VGG16, (**c**) for DenseNet121, (**d**) for FCBTC Model-1, (**e**) for FCBTC Model-2, and (**f**) for FCBTC Model-3.

The confusion matrices for baseline CNN models and FCBTC hybrid Models are given in Figure 9. The color in the confusion matrix represents the magnitude or counts of the cell values. Brighter (lighter) diagonal cells denote higher correct count or percentage. Darker off-diagonal cells denote more frequent misclassifications between specific classes.

#### 3.3.2. Classification Report and Performance Comparison Analysis

From Table 2, it is observed that the hybrid models have outperformed the baseline models.

The overall performance of the FBCTC Model-3 is better than others. It has achieved an accuracy of 98.40% and average precision, recall, and F1-score of 98.33%, 98.26%, and 98.27%, respectively. This highlights the better performance potential of hybrid architectures of CNN models for Brain Tumor classification. The results of other CNN models and our proposed architectures were compared in Table 3.

## 4. Discussion

To build the hybrid architectures, we explored the DenseNet, ResNet, and VGG architectures. The dense connectivity of DenseNet ensures efficient feature propagation while reducing redundancy. The skip connections of ResNet architecture allow residual learning, and the identity mappings enable smooth flow of gradients. The VGG architecture is a simple deep convolutional network model with small 3 × 3 filters to extract features. We trained all the models individually, fine-tuning them to adapt to our domain of brain tumor classification. Then we built hybrid architectures based on our FCBTC framework by combining these three architectures as dual networks, taking two of them at a time. This led us to build a total of six models, three baseline models and three FCBTC framework models. The detailed experimental results of Table 2 show that across all models, the precision and recall scores exceed a minimum of 93.83% for all three types of tumors. For Glioma tumor, the minimum precision is 97.25% for DenseNet121, and the maximum is 100% for FCBTC Model-3; the recall value is a minimum of 94.33% for DenseNet121, and the maximum is 96.67% for FCBTC Model-2. For Meningioma tumors, the minimum precision is 93.83% for DenseNet121, and the maximum is 96.41% for FCBTC Model-2; the recall is a minimum of 94.44% for VGG16 and DenseNet121 and a maximum of 99.02% for FCBTC Model-3. For Pituitary tumor, the minimum precision is 97.38% for VGG16, and the maximum is 99% for DenseNet121 and FCBTC Model-2; the minimum recall is 99% for ResNet50, VGG16, and FCBTC Model-1; and the maximum is 99.33% for FCBTC Model-3.

From Table 3 we see that all our features-concatenated-based architectures have performed superior to other models. The overall Precision, Recall, F1-score, and Accuracy of all our fine-tuned baseline models are also very high. For example, in Table 3, reference [6], the ResNet50 model has an accuracy of 96.5%, but in our case, we have provided the complete architectural design and some modifications that we have made in the pretrained ResNet50 base model mentioned in the Section Fine Tuning, Classifier Modification, and Hybrid Architectural Design. So, in our observation, this modification is accounted for by the ResNet50 model to achieve the accuracy of 97.86%.

For the proposed FCBTC models, the performance surpasses all the previous works. For the FCBTC Model-1, values for Precision, Recall, F1-score, and Accuracy are 97.96%, 97.85%, 97.90%, and 98.02%, respectively. For the FCBTC Model-2, values for Precision, Recall, F1-score, and Accuracy are 98.21%, 98.10%, 98.15%, and 98.25%, respectively. For the FCBTC Model-3, values for Precision, Recall, F1-score, and Accuracy are 98.33%, 98.26%, 98.27%, and 98.40%, respectively. This reinforces our idea that feature diversity does improve the classifier performance.

The study conducted in this research work has its limitations. The dataset used is of Brain MRI scans; hence, the diversity of feature learning is also limited. The study needs to be validated on more diverse datasets like X-ray scans to really prove its usefulness. Further, feature fusion from different layers may also be explored in future studies.

## 5. Conclusions

We introduce the *Features Concatenation-based Brain Tumor Classification (FCBTC) Framework using Hybrid Deep Learning Models* in this work. We explored three baseline models, namely, DenseNet121, ResNet50, and VGG16, and fine-tuned them with modified MLP heads. The hybrid architectures were built by feature concatenation of these baseline models. All the models were trained for 30 epochs on the same Brain MRI images dataset for brain tumor classification. These trained models were evaluated on a testing dataset using standard performance metrics. Among these six models, the hybrid model FCBTC Model-3 (ResNet50 + DenseNet121) has outperformed overall by achieving an accuracy of 98.4% and an average precision, recall, and F1-score of 98.33%, 98.26%, and 98.27%, respectively. This is better than other proposed models and models from different works listed in Table 3. This superior performance can be attributed to the hybrid architecture where two base models have contributed their ability to capture the features, and concatenation of these features has shown better distinct representation of the images, which made the classifier achieve the better performance of classification. These results highlight the potential of hybrid architectures in medical image analysis. In future work, we plan to explore more advanced architectures (e.g., Vision Transformers, attention-based fusion models, and other hybrid architectural enhancements to boost testing performance), evaluation on larger and more diverse datasets, and usage of multi-modal inputs (such as combining imaging with clinical or textual data) to validate scalability.

## Figures and Tables

**Table 1:** Tabular representation of the images’ counts of different tumor classes.

S. No.	Type of Brain Tumor	Number of Images for Training	Number of Images for Testing
1	No Tumor	1595	405
2	Glioma Tumor	1321	300
3	Meningioma Tumor	1339	306
4	Pituitary Tumor	1457	300

**Table 2:** Classification report of all the models for testing dataset.

S. No.	DL Model Type	Tumor Type	Precision (%)	Recall (%)	F1-Score (%)	Accuracy (%)
**1**	**ResNet50 Model**	Glioma	98.28	95	96.61	97.86
		Meningioma	94.59	97.06	95.81	
		No-Tumor	99.51	99.75	99.63	
		Pituitary	98.67	99	98.84	
		**Overall**	**97.76**	**97.70**	**97.72**	
**2**	**Vgg16 Model**	Glioma	98.29	96	97.13	97.56
		Meningioma	95.07	94.44	94.75	
		No-Tumor	99.02	100	99.51	
		Pituitary	97.38	99	98.18	
		**Overall**	**97.44**	**97.36**	**97.39**	
**3**	**DenseNet121 Model**	Glioma	97.25	94.33	95.77	97.25
		Meningioma	93.83	94.44	94.14	
		No-Tumor	98.54	100	99.26	
		Pituitary	99	99.33	99.17	
		**Overall**	**97.16**	**97.03**	**97.09**	
**4**	**FCBTC Model-1** **(VGG16 + Resnet50)**	Glioma	98.97	95.67	97.29	98.02
		Meningioma	95.18	96.73	95.95	
		No-Tumor	99.02	100	99.51	
		Pituitary	98.67	99	98.84	
		**Overall**	**97.96**	**97.85**	**97.90**	
**5**	**FCBTC Model-2 (DenseNet121 + VGG16)**	Glioma	98.64	96.67	97.64	98.25
		Meningioma	96.41	96.41	96.41	
		No-Tumor	98.78	100	99.39	
		Pituitary	99	99.33	99.17	
		**Overall**	**98.21**	**98.10**	**98.15**	
**6**	**FCBTC Model-3 (Resnet50 + DenseNet121)**	Glioma	100	94.67	97.26	**98.40**
		Meningioma	94.98	99.02	96.96	
		No-Tumor	100	100	100	
		Pituitary	98.35	99.33	98.84	
		**Overall**	**98.33**	**98.26**	**98.27**	

**Table 3:** Comparison of Performance results.

Ref.	DL Models	Precision (%)	Recall (%)	F1-Score (%)	Acc. (%)
[2]	CNN Model	-	91.13	-	93.30
[2]	VGG16 Model	-	81.04	-	71.6
[2]	Inception V3 Model	-	79.81	-	80.0
[2]	ResNet50 Model	-	70.03	-	81.1
[3]	ResNet50 Model	-	-	-	96.33
[3]	MobileNet Model	-	-	-	96.94
[3]	MobileNetV2 Model	-	-		94.80
[4]	Tucker decomp. + Extra-Trees Model	-	-	97.16	97.28
[6]	VGG16 Model	97.4	97.7	-	97.6
[6]	ResNet50 Model	96.6	96.8	-	96.5
[9]	CNN Model	-	89.5	91.76	96
[9]	VGG16 Model	-	94.4	92.6	98.15
[31]	CNN Model	95.44	94.67	93.33	97.39
**Proposed work**	Resnet50 Model	97.76	97.70	97.72	97.86
**Proposed work**	VGG16 Model	97.44	97.36	97.39	97.56
**Proposed work**	Denesenet121 Model	97.16	97.03	97.09	97.25
**Proposed work**	FCBTC Model-1 (VGG16 + ResNet50)	97.96	97.85	97.90	98.02
**Proposed work**	FCBTC Model-2 (DeneseNet121 + VGG16)	98.21	98.10	98.15	98.25
**Proposed work**	FCBTC Model-3 (ResNet50 + DeneseNet121)	**98.33**	**98.26**	**98.27**	**98.40**

## Data Availability

The dataset used in this study is publicly available on Kaggle and can be accessed at www.kaggle.com/datasets/masoudnickparvar/brain-tumor-mri-dataset/data. This study did not involve any human data collection; all data used are from a real, publicly archived dataset and were obtained in compliance with open data usage guidelines.
